# The Role of PGC-1α and Mitochondrial Biogenesis in Kidney Diseases

**DOI:** 10.3390/biom10020347

**Published:** 2020-02-24

**Authors:** Miguel Fontecha-Barriuso, Diego Martin-Sanchez, Julio Manuel Martinez-Moreno, Maria Monsalve, Adrian Mario Ramos, Maria Dolores Sanchez-Niño, Marta Ruiz-Ortega, Alberto Ortiz, Ana Belen Sanz

**Affiliations:** 1 Research Institute-Fundacion Jimenez Diaz, Autonoma University, 28040 Madrid, Spain; miguel.fontecha@quironsalud.es (M.F.-B.); diego.martin@fjd.es (D.M.-S.); juliomanuelm@gmail.com (J.M.M.-M.); AMRamos@fjd.es (A.M.R.); mdsanchez@fjd.es (M.D.S.-N.); mruizo@fjd.es (M.R.-O.); AOrtiz@fjd.es (A.O.); 2 REDINREN, 28040 Madrid, Spain; 3 Instituto de Investigaciones Biomédicas “Alberto Sols” (CSIC-UAM), 28029 Madrid, Spain; mpmonsalve@iib.uam.es; 4 School of Medicine, UAM, 28029 Madrid, Spain; 5 IRSIN, 28040 Madrid, Spain

**Keywords:** kidney, mitochondrial biogenesis, sirtuin, PGC-1α, oxidative stress, diabetes, acute kidney injury

## Abstract

Chronic kidney disease (CKD) is one of the fastest growing causes of death worldwide, emphasizing the need to develop novel therapeutic approaches. CKD predisposes to acute kidney injury (AKI) and AKI favors CKD progression. Mitochondrial derangements are common features of both AKI and CKD and mitochondria-targeting therapies are under study as nephroprotective agents. PGC-1α is a master regulator of mitochondrial biogenesis and an attractive therapeutic target. Low PGC-1α levels and decreased transcription of its gene targets have been observed in both preclinical AKI (nephrotoxic, endotoxemia, and ischemia-reperfusion) and in experimental and human CKD, most notably diabetic nephropathy. In mice, PGC-1α deficiency was associated with subclinical CKD and predisposition to AKI while PGC-1α overexpression in tubular cells protected from AKI of diverse causes. Several therapeutic strategies may increase kidney PGC-1α activity and have been successfully tested in animal models. These include AMP-activated protein kinase (AMPK) activators, phosphodiesterase (PDE) inhibitors, and anti-TWEAK antibodies. In conclusion, low PGC-1α activity appears to be a common feature of AKI and CKD and recent characterization of nephroprotective approaches that increase PGC-1α activity may pave the way for nephroprotective strategies potentially effective in both AKI and CKD.

## 1. AKI, CKD and the Mitochondrial Connection

Kidney diseases represent a growing worldwide burden and may cause acute kidney injury (AKI) and/or chronic kidney disease (CKD). AKI is characterized by a rapid, often reversible loss of renal function. Current AKI mortality remains around 50% [1]. There is no effective treatment for AKI that accelerates recovery. The only treatment is symptomatic and consists of replacement of renal function by dialysis in severe cases [2]. Key causes of AKI include sepsis, ischemia-reperfusion injury (IRI) and nephrotoxic agents [2,3]. 

The first events during AKI, sublethal cell injury, cell death, and inflammation, are followed by regeneration leading to kidney function recovery, but suboptimal regeneration may result in transition to CKD [4]. The initial wave of tubular cell death is followed by tubular cell proliferation favoring regeneration, and by a second wave of cell death that adjusts the final cell number [5,6]. Regulated necrosis cell death through ferroptosis and necroptosis releases inflammatory cytokines that amplify tissue injury [7,8] while there is also evidence for a contribution of apoptotic cell death to kidney injury [9]. 

CKD is among the fastest growing global causes of death; it is projected to become the fifth global cause of death by 2040 and the second cause of death in long-lived countries before the end of the century [10,11]. CKD is usually irreversible and is defined by decreased kidney function assessed by glomerular filtration rate (GFR, <60 mL/min per 1.73 m^2^), or evidence of kidney injury, such as pathological albuminuria (>30 mg/g of urinary creatinine) persisting for at least 3 months [12,13]. End-stage kidney disease requires kidney function replacement by dialysis or kidney transplantation [14]. Therapeutic options to prevent CKD progression are limited, contributing to the high burden of disease. Thus, it is imperative to develop new therapeutic options for AKI and CKD. Research approaches may take advantage of the fact that AKI and CKD are interconnected syndromes to target common pathogenic pathways [15,16,17,18]. Thus, AKI episodes favor CKD progression, and CKD is a risk factor of AKI. A key role of mitochondria in kidney injury has been suggested by interventional studies [19]. In fact, mitochondrial dysfunction is a key factor in the pathogenesis of AKI, causing tubular cell dysfunction and death [20,21]. Mitochondrial homeostasis results from fusion or fission of pre-existing mitochondria, degradation of dysfunctional mitochondria via mitophagy or the ubiquitin proteasome system, and generation of new mitochondria from existing mitochondria via mitochondrial biogenesis [22,23]. We now review the contribution to kidney disease of the altered expression and activity of a key transcription factor in mitochondrial biogenesis: Peroxisome proliferator-activated receptor-γ coactivator-1-α (PGC-1α). Specifically, after an overview of the function of PGC-1α in mitochondrial biogenesis, its role in health and disease, and the involvement of mitochondria in kidney disease, we review evidence supporting the concept that PGC-1α derangements contribute to kidney disease and can be therapeutically targeted.

## 2. PGC-1α: A Regulator of Mitochondrial Biogenesis

**Figure 1:**
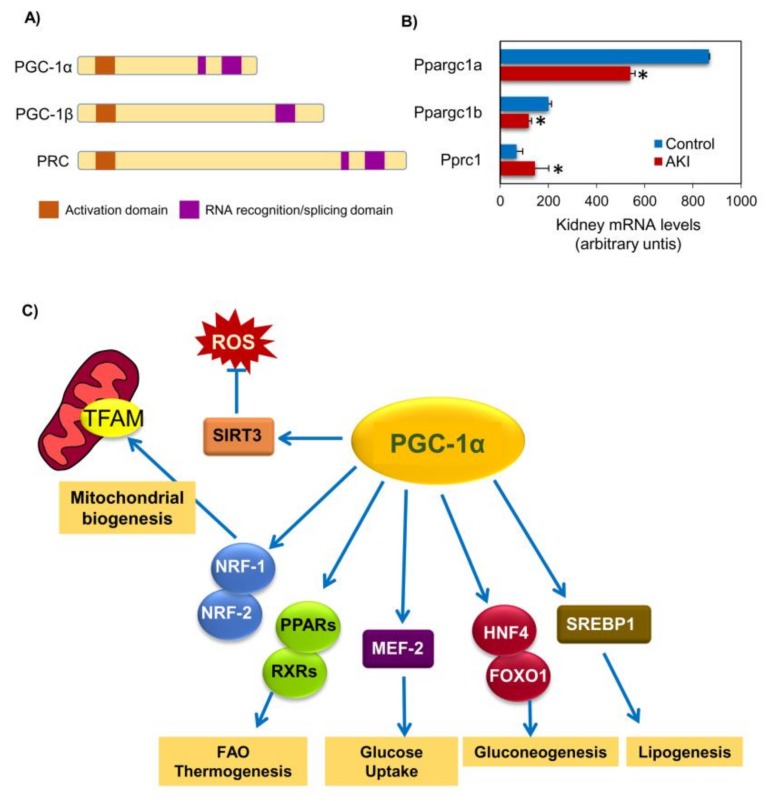
Peroxisome proliferator-activated receptor-γ coactivator-1-α (PGC-1α) structure, family and functions. (**A**) Structure of the PGC-1α family of transcriptional regulators. (**B**) Gene expression of the PGC-1α family of transcriptional regulators during experimental folic acid-induced acute kidney injury (AKI) [28]. * False discovery rate <0.02 vs control. (**C**) Key PGC-1α functions. Although in the context of kidney disease and this review we will focus on the role of PGC-1α in mitochondrial biogenesis, it has multiple additional functions in metabolism homeostasis that involve different binding partners regulating gene transcription as well as multiple target genes and proteins. Abbreviations: FAO: fatty acid β-oxidation, FOXO1: fork head box O1, HNF-4: hepatic nuclear factor-4, MEF-2: myocyte enhancer factor-2, NRF-1/2: nuclear respiratory factor 1/2, PPARs: peroxisome proliferator-activated receptors, PRC: PGC-1-related coactivator, ROS: reactive oxygen species, RXR: retinoid receptors, Sirt3: sirtuin 3, STREBP1: sterol regulatory element-binding protein 1, TFAM: mitochondrial transcription factor A.

Mitochondrial biogenesis is a complex process that involves synthesis of the inner and outer mitochondrial membranes and mitochondrial encoded proteins synthesis and import of nuclear encoded mitochondrial proteins and replication of mitochondrial DNA (mtDNA), requiring a coordinated regulation of two distinct genomes, the nuclear and mitochondrial genomes, given that a majority of mitochondrial proteins are encoded by nuclear genes [22,23]. PGC-1α is a master regulator of mitochondrial biogenesis that coordinates the transcriptional machinery leading to increased mitochondrial mass thus allowing tissue adaptation to increased energetic demands [23]. PGC-1α is encoded by the *PPARGC1A* gene and belongs to the PGC-1 family, also composed of PGC-1β (encoded by *PPARGC1B*), which contributes to maintain basal mitochondrial function, and PRC (PGC-1-related coactivator, encoded by *PPRC1*), which appears to be restricted to regulating mitochondrial biogenesis in proliferating cells [24] (Figure 1A). While there is abundant literature on PGC-1α and kidney disease, as discussed below, much less is known about the role of PGC-1β and PRC in kidney disease, despite the fact that transcriptomic studies have identified them as differentially expressed during experimental AKI [25,26,27] (Figure 1B).

**Figure 2:**
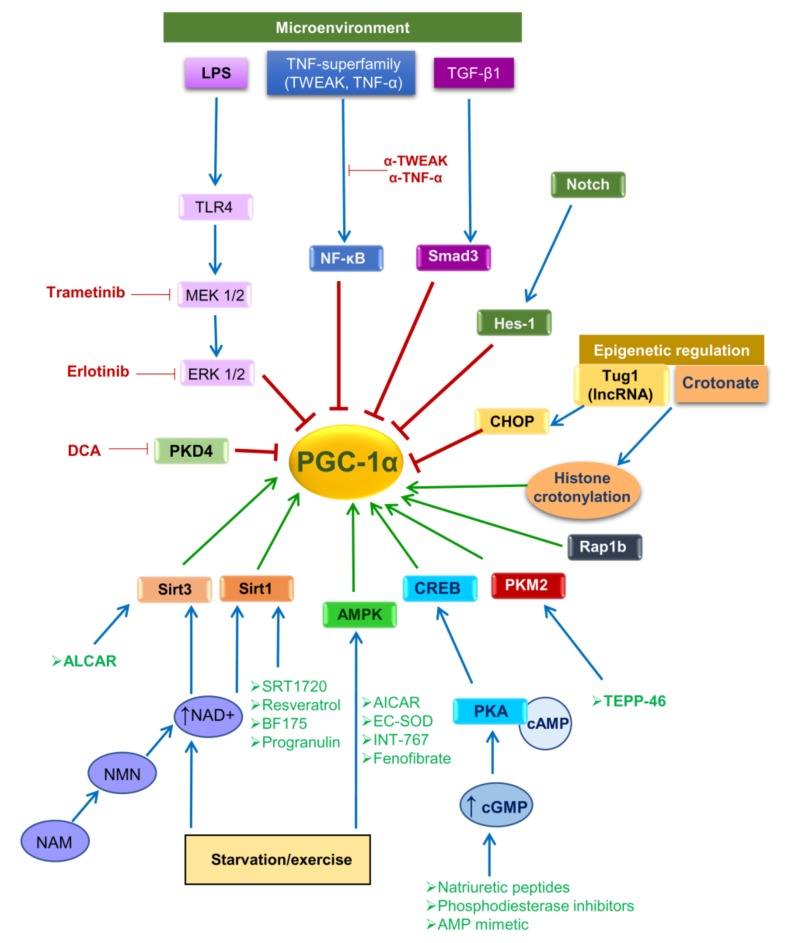
Regulators of PGC-1α gene expression, activity and potential for therapeutic intervention aimed at increasing PGC-1α activity. The microenvironment (including mediators of inflammation and fibrosis), starvation/exercise and epigenetic regulation are the main modulators of PGC-1α activity. These, in turn, modulate intracellular signaling pathways that negatively (red lines) or positively (green lines) regulate PGC-1α gene expression and activity. Therapeutic approaches potentially upregulating PGC-1α gene expression and activity are depicted outside boxes: in green, those that activate specific intracellular signaling pathways to increase PGC-1α activity, and in red, those that inhibit specific intracellular signaling pathways to promote PGC-1α activity. Abbreviations: AICAR: 5-aminoimidazole-4-carboxamide-1-β-d-ribofuranoside, ALCAR: antioxidant agent acetyl-l-carnitine, AMPK: AMP-activated protein kinase, cAMP: cyclic adenosine monophosphate, cGMP: cyclic guanosine monophosphate, CHOP: C/EBP homologous protein, CREB: cAMP responsive element binding protein, DCA: dichloroacetate, ERK: extracellular signal-regulated kinase, LPS: lipopolysaccharide, MEK: mitogen-activated protein kinase kinase, NAD: nicotinamide adenine dinucleotide, NAM: niacinamide, NF-κB: nuclear factor κB, NMN: nicotinamide mononucleotide, PDK4: pyruvate dehydrogenase kinase 4, PKA: protein kinase A, PKM2: pyruvate kinase M2, Rap1b: RAS-Related Protein Rap1b, Sirt1: sirtuin 1, Sirt3: sirtuin 3, TEPP-46: tetraethyl diphosphate 46, TGF-β1: transforming growth factor β1, TLR4: toll-like receptor 4, TNF-α: tumor necrosis factor α, Tug1: taurine up-regulated gene 1, TWEAK: TNF-like weak inducer of apoptosis.

PGC-1α activity is regulated by both posttranslational modifications and by gene expression levels (Figure 2). PGC-1α can be activated by posttranslational modifications (e.g., phosphorylation or de-acetylation) or by increased transcription. Stressors (glucose deprivation, starvation or exercise) lead to AMP-activated protein kinase (AMPK)-mediated phosphorylation of PGC-1α as well as to increased nicotinamide adenine dinucleotide (NAD) levels resulting in activation of Sirtuin-1 (SIRT1), a NAD-dependent deacetylase which deacetylates PGC-1α. In preclinical studies, increased NAD biosynthesis reduces renal injury, but the mechanism is unclear given the multiple functions of NAD [29,30,31]. Active PGC-1α translocates into the nucleus where it activates nuclear respiratory factor 1 and 2 (Nrf1 and Nrf2), and subsequently the transcription of nuclear coded respiratory chain components and mitochondrial transcription factor A (Tfam) thus promoting synthesis of mitochondrial proteins, mtDNA replication and transcription, and new mitochondria biogenesis [22,23] (Figure 1C). PGC-1α may also interact with additional nuclear factors, thus regulating multiple pathways involved in cellular energy metabolism within and outside mitochondria. The list of partners includes PPARα, PPARβ, retinoid receptors (RXR), myocyte enhancer factor-2 (MEF-2), fork head box O1 (FOXO1), hepatic nuclear factor-4 (HNF-4), sterol regulatory element-binding protein 1 (SREBP1) or estrogen-related receptors (ERRs), among others (Figure 1C). These transcription factors regulate genes involved in mitochondrial fatty acid oxidation, lipogenesis, thermogenesis, and glucose metabolism [32], which are critical for tissues with high metabolic demands, such as heart, skeletal muscle, brain and kidney [22]. 

PGC-1α mRNA levels also have positive and negative regulators (Figure 2). At the transcriptional level, cAMP response element-binding protein (CREB) activates PGC-1α transcription [22,33]. High cAMP or cGMP levels activate protein kinase A (PKA) which in turn, phosphorylates and activates CREB. cGMP is a key regulator of mitochondrial biogenesis and cGMP-specific phosphodiesterase (PDE) inhibitors, which increase cGMP by blocking its cleavage to GMP, promote PGC-1α expression and mitochondrial biogenesis in the heart and kidney [34,35]. Moreover, natriuretic peptides also stimulate cGMP synthesis and promote PGC-1α expression and mitochondrial biogenesis [36].

In contrast, inflammatory mediators such as TNF-α or TWEAK reduce PGC-1α expression and mitochondrial biogenesis through activation of nuclear factor-κB (NF-κB) and epigenetic regulation [37,38]. Furthermore, profibrotic factors may also reduce PGC1α expression. The profibrotic transcription factors Hes1, a downstream target of Notch signaling [39], can directly bind the PGC-1α promoter region and inhibit PGC-1α gene expression. Moreover, TGF-β1 decreases PGC-1α levels though epigenetic downregulation via Smad3 [40].

## 3. PGC-1α in Health and Disease

A role for PGC-1α has been described in diabetes and pancreatitis, liver disease, and endothelial cell injury, in addition to kidney disease.

### 3.1. Diabetes

PGC-1α is expressed in liver and pancreatic β cells, two key players in diabetes mellitus (DM). Liver PGC-1α is induced by glucagon and other mediators that increase intracellular cAMP levels and CREB activation [41]. In the liver, PGC-1α not only controls mitochondrial biogenesis and fatty acid oxidation, as in other organs but also drives fasting-induced gluconeogenesis [42]. Indeed, liver and pancreas PGC-1α expression is increased in diabetic patients and animals [43]. However, the role of PGC-1α in diabetes is still controversial, possibly due to the different functions of PGC-1α in different organs [44]. In pancreatic β-cells, forced PGC-1α expression inhibits glucose-stimulated insulin secretion in vitro and in vivo [45] and PGC-1α activity drives β-cell apoptosis in response to high glucose levels [46], suggesting that PGC-1α may favor DM development. The proposed mechanism was increased expression of uncoupling protein-2 (UCP-2), which is known to reduce glucose-stimulated insulin secretion [47,48]. UCP-2 is expressed under conditions driving mitochondrial dysfunction, including hyperglycemia, and reduces the mitochondrial membrane potential when the electron flow cannot be coupled to ATP synthesis. PGC-1α overexpression may also inhibit β-cell differentiation [49,50]. However, human genetic studies suggest a protective role of PGC-1α in DM [51]. Thus, Gly482Ser, the most common *PPARGC1A* polymorphism, is associated with Type 2 DM (T2DM), but results in decreased PGC-1α mRNA levels and insulin secretion [52] and insulin resistance [53]. In this regard, high glucose and palmitic acid (a key mediator of β-cell lipotoxicity) concentrations down-regulate PGC-1α levels [54,55] and inducible PGC-1α deletion in β-cells results in decreased insulin secretion [56]. These results suggest a general protective role of PGC-1α, that might be lost under disease conditions, and also, a tight regulation of the system in which excess inappropriate PGC-1α may be deleterious. The understanding of these relationships is key to developing PGC-1α-based therapeutic approaches for kidney disease since diabetic nephropathy which is the most frequent cause of CKD, and also predisposes to AKI [57]. In this regard, metabolomics identified a signature of mitochondrial dysfunction in human diabetic nephropathy, associated with lower PGC-1α gene expression and is evidence of an overall impaired mitochondrial biogenesis [58,59] (discussed below). 

### 3.2. Pancreatitis

PGC-1α protects the pancreas from the complications of acute pancreatitis, which is more frequent and has poorer outcomes in obese subjects who have low pancreas PGC-1α levels. Thus, PGC-1α deficient mice were more sensitive to acute pancreatitis induced by cerulein due to a reduced capacity to control the resulting inflammatory response, leading to an uncontrolled over-activation of NF-κB and the subsequent induction of IL-6 [60].

### 3.3. Liver Disease

PGC-1α deficient mice are insulin sensitive and are not hypoglucemic in normal conditions but, when fasted, fail to induce gluconeogenesis and accumulate lipids in the liver, leading to liver steatosis [61]. Accordingly, PGC-1α levels are reduced in liver steatosis, a common condition that is a risk factor for liver disease and that yields transplanted livers more sensitive to IRI [62,63,64]. Loss of PGC-1α is a key factor in the enhanced susceptibility of steatotic livers to IRI and PGC-1α activity is necessary for ischemic preconditioning [65]. This effect is likely associated with the induction of antioxidant gene expression by PGC-1α. Similarly, PGC-1α protects from alcoholic and non-alcoholic fatty liver disease, from viral-induced steatohepatitis and from hepatotoxicity [66,67,68,69,70]. These protective effects may be related at least in part to the negative regulation of liver inflammation by PGC-1α. Importantly, in the damaged, inflamed liver, PGC-1α levels are further downregulated by inflammatory mediators like TNF-α [71].

Another liver-specific activity of PGC-1α is regulation of Selenoprotein P (SeP), which controls selenium homeostasis [72]. Selenium is a cofactor of selenoproteins that play key roles in cellular redox control [73]. In this regard, human livers express a liver-specific PGC-1α transcript (L-PGC-1α) resulting from using an alternative promoter [74]. While coactivation properties mostly overlap with the ubiquitous PGC-1α, there are functional differences. For example, L-PGC-1α seems unable to coactivate liver X receptor alpha (LXRα).

While traditionally the hepatorenal syndrome causing AKI was the main kidney-related concern in liver disease patients, more recently a link between liver steatosis, non-alcoholic fatty liver disease (NAFLD) and CKD has been emphasized [75,76]. Since NAFLD, diabetes and CKD are complications of the metabolic syndrome, this points to the potential utility of PGC-1α-based therapeutic approaches to target the different complications of metabolic syndrome.

### 3.4. Endothelium

Endothelial cells are generally regarded as glycolytic cells that make a very limited use of mitochondria. However, they do express PGC-1α that in these cells regulates antioxidant gene expression. Thus, PGC-1α prevented high glucose-induced endothelial dysfunction and increased eNOS expression and the synthesis of NO synthesis, a key modulator of vascular relaxation [77]. In fact, NO itself regulates endothelial PGC-1α expression [78]. PGC-1α may also protect from hyperlipidemia-induced endothelial dysfunction. Thus, C1q/TNF-Related Protein-9 protects from Oxidized-LDL-induced endothelial dysfunction via PGC-1α [79]. 

Loss of endothelial PGC-1α induced a mesenchymal transition characterized by reduced adhesion, increased migration, cytoskeletal disorganization and poor adherence junctions resulting in an exacerbated tip cell-like phenotype that responded poorly to angiogenic mediators, such as vascular endothelial growth factor A (VEGF-A) [80,81]. Tip cells at the end of developing blood vessels drive vessel formation. PGC-1α-deficient mice had a significant loss of pericyte coverage of retinal endothelial cells. Pericytes normally maintain vascular structures and pericyte loss correlates with reduced vascular perfusion [82]. In this regard, PGC-1α deficient mice developed more severe hyperoxia-induced retinal vascular abnormalities and retinopathy [82]. These results therefore suggest that PGC-1α may be protective in microvascular diseases, including retinopathies. In line with these results, PGC-1α heterozygous mice exposed to a high fat diet developed age-related macular degeneration-like abnormalities in the retinal pigmented epithelium and local inflammatory responses [83]. In the context of kidney diseases, endothelial dysfunction associated with hypertension, obesity and/or diabetes is a key mechanism in CKD progression and CKD-related cardiovascular complications [84,85,86,87]. Thus, prevention of endothelial injury may also be beneficial in the kidney disease context. In this regard, diabetic nephropathy is considered a manifestation of diabetic microvascular disease.

## 4. Mitochondria in Kidney Diseases

Due to the high demand for energy for solute reabsorption [88], the kidneys, and particularly proximal tubular and medullary thick ascending limb cells display a high mitochondrial density [89]. The main source of energy in the kidney is ATP generation from fatty acid β-oxidation (FAO) in tubular cell mitochondria, a process regulated by carnitine palmitoyl-transferase 1 (CPT1) as the limiting enzyme. Tubular cells are very sensitive to kidney insults, and mitochondria are key players in cell death, particularly in apoptotic cell death [90]. Additionally, mitochondria may get damaged and dysfunctional in the course of kidney injury. In this regard, there is increasing functional and interventional evidence supporting a key role for mitochondria in kidney disease.

### 4.1. Morphological and Functional Changes of Mitochondria

Kidney injury may alter mitochondrial structure and function in different manners, including alterations of calcium homeostasis, membrane integrity, ROS production, mitochondrial transport, biogenesis, dynamics (fusion/fission) and mitophagy [91,92,93]. Altered mitochondrial homeostasis may in turn further promote kidney injury triggering a harmful feedback loop [19,92,94,95,96]. 

Mitochondrial injury plays a key role in experimental AKI models triggered by sepsis [94,97,98], IRI [99,100,101,102,103], and nephrotoxicity [104,105,106]. Mitochondrial damage in AKI is associated with mitochondrial fragmentation [107], reduced mitochondrial mass [108], mitochondrial swelling and cristae disruption [109,110], apoptosis [111], and, in general, with impaired mitochondrial function [104,105,106]. Mutations or large deletions on mtDNA or nuclear genes encoding for mitochondrial proteins may result in kidney cysts or glomerular or tubular disease [112,113,114,115,116]. CKD and kidney tumors have also been linked to mitochondrial abnormalities. Dysmorphic mitochondria, reduced mtDNA [117] and decreased ATP production by mitochondria contribute to the development and progression of diabetic nephropathy [118,119] while chronic allograft nephropathy is characterized by reduced mitochondrial biogenesis, inadequate energy production and impaired antioxidant function [120]. In human CKD, lower renal expression of key FAO enzymes (such as CPT1 and 2), transcription factors (peroxisome proliferator-activated receptors) and other mitochondrial genes are associated with kidney fibrosis [121], suggesting a dysregulated cell metabolism in CKD. In this sense, transgenic and pharmacological approaches that restore FAO were beneficial in experimental kidney damage [121]. In CKD, tubular cells may undergo partial epithelial-to-mesenchymal transition and generate a senescence-like secretome that contributes to CKD progression. Senescence may be associated with mitochondrial injury and increased mitophagy [122,123]. Additionally, both defective DNA repair and early low renal mitochondrial activity are associated with benign renal tumors and renal cell carcinoma [124,125,126,127].

### 4.2. Mitochondria-Targeted Therapies

Emerging evidence suggests that mitochondria-targeted therapies acting upstream of cellular damage could present advantages over targeting downstream processes, such as inflammation and fibrosis. Novel therapeutic strategies for mitochondrial disease are being developed in preclinical studies and are entering human clinical trials [128,129,130]. Mitochondria-targeted therapies aim to enhance mitochondrial function, acting on electron transfer chain (ETC) function, mitochondrial biogenesis or promoting FAO, or to dampen the cellular consequences of mitochondrial dysfunction, including ROS production, inflammasome activation, apoptosis, pyroptosis, and autophagy/mitophagy. Treatments that enhance ETC components, and therefore electron transfer (CoQ10, idebenone, and riboflavin) or increase ETC substrate availability (dichloroacetate, and thiamine) have been tested in mitochondrial diseases [128]. Mitochondria-targeted therapies were nephroprotective in experimental AKI [19]. Initial approaches used mitochondrial permeability transition pore inhibitors [19]. More recent strategies are aimed at restoring mitochondrial metabolism, redox state, and dynamics, as well as enhancing biogenesis [131,132].

Mitochondrial-targeted antioxidants, including the Szeto–Schiller (SS) peptides, MitoQ, and plastoquinone analogues (SkQ1/SkQR1), accumulate within the mitochondrial matrix and interact with cardiolipin, a major constituent of the mitochondrial inner membrane [133]. SS peptides promote ATP synthesis, reduce electron leak and ROS production, and inhibit cardiolipin peroxidation [134], preventing the consequences of mitochondrial dysfunction, including apoptosis, inflammation, and NLRP3 inflammasome activation [134]. Specifically, SSP3 protected from experimental AKI [135] and in kidney injury in T1DM induced by streptozotocin in rats [136]. MitoQ, MitoTEMPO, or SkQR1 also reduced oxidative damage and renal inflammation [134,137,138,139]. MitoQ protected from diabetes-induced CKD in T2DM db/db and in T1DM Ins2±AkitaJ mice however, in the latter model, mitochondrial function was reported to be normal [138,140].

Finally, miRNAs targeting fibrosis-associated genes (like miR21 or miR9) were protective by inducing metabolic reprograming mainly through modulation of mitochondrial-damage related genes [141,142].

## 5. PGC-1α in Kidney Diseases

Evidence for the contribution of decreased PGC-1α levels or activity to the pathogenesis of AKI is derived from in vivo animal studies. The role of PGC-1α in experimental kidney diseases has been explored in mice which overexpressed PGC-1α or had a PGC-1α genetic deficiency. PGC-1α-KO mice had normal renal function assessed by serum creatinine [28,94] although there was evidence of subclinical kidney injury characterized by tubulointerstitial inflammation, increased NGAL expression and oversensitivity to AKI [28]. Additionally, there is indirect evidence based on the use of PGC-1α activators for a protective role of PGC-1α in experimental CKD.

### 5.1. PGC-1α in AKI

**Figure 3:**
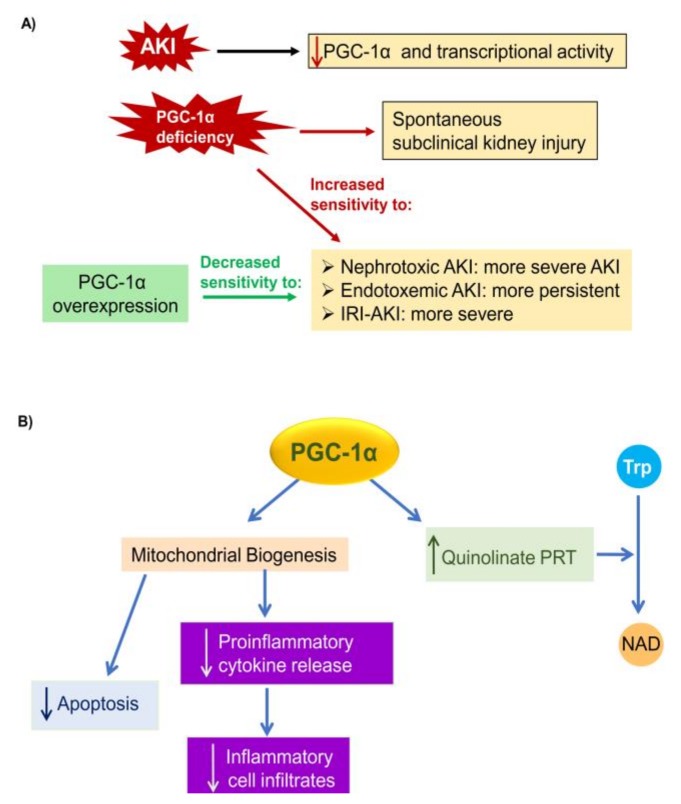
**PGC-1α and AKI**. (**A**) AKI is characterized by a decrease in PGC-1α gene expression and activity leading to downregulation of PGC-1α target genes. Genetic PGC-1α deficiency is associated with spontaneous evidence of subclinical CKD as well as with increased sensitivity to AKI (more severe and/or more persistent as shown in the figure). PGC-1α overexpression in tubular cells is associated with decreased severity of experimental AKI. Additionally, interventions that result in increased PGC-1α gene transcription or activity are also associated with decreased AKI severity. (**B**) Nephroprotective actions of PGC-1α in tubular cells. Pathways are shown that are responsive to higher PGC-1α levels in tubular cells and/or AKI, and to contribute to nephroprotection. Abbreviations: Trp: tryptophan, NAD: nicotinamide adenine dinucleotide.

There is functional *in vivo* evidence of the role of insufficient PGC-1α activation or even PGC-1α downregulation in the pathogenesis of diverse forms of AKI, as well as of the therapeutic impact of preserving PGC-1α expression in experimental AKI (Table 1, Figure 3A). Tubular cells are key cell types in AKI and PGC-1α was protective in these cells (Figure 3B).

**Nephrotoxic AKI.** The role of PGC-1α in nephrotoxic AKI has been studied in experimental folic acid-induced AKI (FA-AKI) and cisplatin nephrotoxicity. PGC-1α mRNA expression and protein levels decrease within 24h in FA-AKI and upstream regulator analysis of kidney transcriptomic data identified PGC-1α as the transcriptional regulator whose activity is most dramatically reduced in AKI [28,37,143]. The repression of PGC-1α activity appeared to be functionally relevant given the downregulated gene expression of PGC-1α canonical targets such as electron transport chain components and TFAM leading to reduced mitochondrial biogenesis. Proinflammatory cytokines such as TWEAK were identified as key drivers of PGC-1α downregulation during AKI. In FA-AKI, neutralizing anti-TWEAK antibodies prevented the kidney downregulation of PGC-1α and its targets. In addition, systemic TWEAK administration decreased PGC-1α expression in healthy kidneys and in cultured tubular cells this decrease was prevented by NF-κB inhibitors [37]. Crotonylation, a post-translational modification of histones [144], may also regulate PGC-1α expression. Thus, crotonate, a precursor of the substrate for histone crotonylases, reduced renal injury and increased PGC-1α expression in FA-AKI [145]. However, it is unclear whether modulation of PGC-1α expression was the only or even the main driver of the nephroprotection offered by crotonate.

PGC-1α overexpression and genetic depletion approaches further support its role in nephrotoxic AKI. In cultured tubular cells, adenoviral-mediated PGC-1α overexpression prevented TWEAK-induced downregulation of PGC-1α-dependent genes and the decrease in mitochondrial membrane potential [37]. Indeed, over-expression of PGC-1α in renal proximal tubule cells restored mitochondrial and cellular functions after oxidant exposure, demonstrating the role of mitochondrial biogenesis in recovery from cellular injury [146]. 

PGC-1α-KO mice with FA-AKI had increased mortality and more severe loss of renal function and tubulointerstitial injury (tubular cell death and compensatory proliferation, expression of pro-inflammatory cytokines, NF-κB activation and interstitial inflammatory cell infiltration). This was associated with decreased kidney expression of mitochondrial PGC-1α-dependent genes and an earlier decrease in mitochondrial mass than in wild type (WT) mice. Thus, PGC-1α KO mice had lower mitochondrial biogenesis. The more severe inflammation in PGC-1α KO mice was characterized by increased M1 and decreased M2 responses and lower expression of the anti-inflammatory cytokine IL-10 [28]. In cultured renal tubular cells, PGC-1α targeting promoted cell death and pro-inflammatory responses [28].

Selective inhibitors of cGMP-specific PDE increased PGC-1α expression and mitochondrial biogenesis in cultured renal tubular cells, in healthy kidney and in kidneys with FA-AKI [34]. However, their effect on renal function and renal injury was not well characterized.

Nephrotoxicity is the dose-limiting adverse effect of cisplatin and interventional studies suggest that mitochondrial injury may play a critical role, as different strategies to preserve mitochondrial function were nephroprotective [106,147,148,149,150,151]. Indeed, cisplatin downregulates kidney tubular PGC-1α *in vivo* and in cultured tubular cells [152,153,154]. Pyruvate dehydrogenase kinase 4 (PDK4) plays a key role in cisplatin-induced AKI, since PDK4 deficiency prevented AKI and the decrease in PGC-1α expression and mitochondrial biogenesis [154]. The AMPK activator 5-aminoimidazole-4-carboxamide-1-β-d-ribofuranoside (AICAR) and the antioxidant agent acetyl-l-carnitine (ALCAR), activate SIRT3, another member of the sirtuin family of NAD-dependent deacetylases. Both drugs increased PGC-1α expression and decreased mitochondrial fragmentation and renal function in experimental cisplatin-induced AKI, while SIRT3 deficiency increased AKI severity [155]. This fits well with the positive feedback loop between PGC-1α and SIRT3 expression [145,156]. As commented above, PGC-1α regulates NAD biosynthesis. Interestingly, the NAD precursor nicotinamide mononucleotide (NMN) increased NAD biosynthesis and protected from cisplatin nephrotoxicity and this was linked to SIRT1 [29,31,157]. However, as PGC-1α is a SIRT1 target, we cannot discard that PGC-1α modulation is required for NAD nephroprotection. 

**Sepsis-associated AKI (s-AKI**)**.** s-AKI is frequent in critically ill sepsis patients and is associated with a high mortality [158,159]. Experimental models reproduce s-AKI by cecal puncture ligation (a bacteremia model closer to the clinical situation) or by administering bacterial lipopolysaccharide (LPS), inducing endotoxemia and a sterile systemic inflammatory response.

s-AKI is characterized by tubular cell death, interstitial inflammatory cell infiltration and mitochondrial dysfunction [160]. Thus, swollen mitochondria were observed in human and experimental s-AKI tubules [94,161,162]. During sepsis, kidney oxygen consumption is reduced leading to decreased intracellular ATP levels, but this is not due to a reduced tissue oxygenation [94,163]. Rather, in murine LPS-induced AKI, kidney mitochondrial dysfunction, and reduced expression of PGC-1α and its mitochondrial target genes correlated with AKI severity [94,162]. As it was the case for FA-AKI, inflammatory cytokines are thought to decrease kidney PGC-1α in s-AKI, as TNF-α decreased PGC-1α expression in cultured renal tubular cells, leading to mitochondrial dysfunction, and also decreased kidney PGC-1α upon systemic administration in vivo [162]. Additionally, LPS itself directly activates TLR4 to downregulate PGC-1α via engagement of the transcription factor NF-κB in cultured cells [37,164,165], but whether NF-κB targeting in vivo preserves PGC-1α expression in s-AKI was not explored in detail. However, the role of MAPK was characterized and the MEK1/2 (MAP2K1/MAP2K2) inhibitor GSK1120212 prevented renal injury and PGC-1α downregulation following LPS injection [162]. This is consistent with prior data showing that ERK1/2 activation regulated PGC-1α expression in tissues such as brain and skeletal muscle [165,166].

Tubule-specific PGC-1α-knockout mice suffered persistent kidney injury following endotoxemia [94]. By contrast, enforced PGC-1α expression in human tubular proximal epithelial cells prevented TNF-induced mitochondrial injury, but the *in vivo* impact on kidney injury was not studied [94]. During sepsis, mechanisms that might restore PGC-1α expression are also activated, such as AMPK and sirtuins [167]. An improved understanding of these compensatory mechanisms may help design therapeutic approaches to preserve PGC-1α levels during s-AKI. PGC-1α expression and mitochondrial biogenesis could also be regulated by miRNAs, at least in adipocytes and skeletal muscle [168,169]. Differentially expressed serum miRNAs from s-AKI patients targeted oxidative stress and mitochondrial functions, and miR-4270, a PGC-1α regulator, was upregulated in serum of s-AKI patients [170]. This opens a new window to preserve PGC-1α expression but further studies are necessary to define the exact role of specific miRNAs in s-AKI and in mitochondrial dysfunction.

**Ischemia-reperfusion injury**. Kidney IRI is a frequent cause of AKI after major surgery and following kidney transplantation that is characterized by PGC-1α downregulation and mitochondrial dysfunction [31,171,172,173,174,175,176]. Kidney PGC-1α is reduced within 24 hours of kidney IRI, and PGC-1α-deficient mice had more severe AKI and accumulated fatty acids in tubular cells. This was ascribed to PGC-1α regulation of the expression of enzymes required for de novo NAD synthesis from tryptophan, and more specifically, of quinolinate phosphoribosyl transferase [30,31]. Thus, two NAD precursors (niacinamide (NAM) and NMN) were nephroprotective in murine IRI, and a phase 1 pilot study of NAM administration to patients undergoing cardiac surgery showed promising results on AKI incidence [29,30,31].

By contrast, specific tubular PGC-1α overexpression increased survival after kidney IRI, and reduced tubular injury and fatty acid accumulation [31]. This suggests that preservation of kidney PGC-1α may be protective in kidney IRI as well as for s-AKI. In this regard, strategies that increase PGC-1α expression were protective in kidney IRI. Both caloric restriction and SRT1720, which activate AMPK and Sirt1 respectively, activated PGC-1α and were protective in rat IRI-AKI and decreased kidney inflammation [175,176]. Further studies are needed to test if nephroprotection led to increased PGC-1α expression or was a consequence of increased PGC-1α [175,176]. In this regard, formoterol, a β2-adrenergic receptor agonist, decreased the severity of IRI-AKI and increased the expression of mitochondrial proteins but had a weak effect on PGC-1α expression.

### 5.2. PGC-1α in CKD

The role of PGC-1α in CKD and potential therapeutic approaches to preserve PGC-1α activity has been most extensively studied in diabetic nephropathy, although additional forms of CKD characterized by kidney fibrosis have also been studied.

**Diabetic nephropathy.** Given that diabetic nephropathy is proteinuric and characterized by mesangial extracellular matrix deposition, most studies have explored podocytes and mesangial cells, although it is likely that tubular cells are even more compromised from a mitochondrial point of view. However, the contribution of mitochondrial derangement and, specifically, podocyte PGC1α deficiency to podocyte physiology has been questioned [177]. Indeed, while PGC-1α overexpression may be beneficial in cultured tubular cells, forced PGC-1α overexpression in podocytes caused collapsing glomerulopathy, suggesting that a tight regulation in at least some cell types is required [178]. In any case, the study of diabetic nephropathy has identified PGC-1α regulators of potential therapeutic interest. Thus, PGC-1α activators were nephroprotective in both in experimental T1DM and T2DM.

Glomerular SIRT1 expression is reduced in human diabetic glomeruli; the podocyte-specific loss of SIRT1 increased albuminuria and accelerated kidney disease progression in T1DMc OVE26 mice. The selective SIRT1 agonist BT175 increased SIRT1-mediated activation of PGC1-α and protected against high glucose-mediated mitochondrial injury in cultured podocytes while both podocyte-specific SIRT1 overexpression and BT175 decreased diabetes-induced podocytopenia and glomerular oxidative stress in mice [179]. Resveratrol, a natural polyphenolic antioxidant and SIRT1 agonist, restored renal SIRT1 and PGC-1α levels in both T2DM db/db mice and in mice with T1DM induced by streptozotocin [180,181]. Resveratrol also increased PGC-1α expression and decreased apoptosis and mitochondrial oxidative stress in podocytes exposed to high glucose [180,181]. In addition, in db/db mice, inhibition of TLR4/NF-κB signaling prevented the decreased kidney PGC-1α expression, mitochondrial dysfunction and deformation, and ROS accumulation, while PGC-1α overexpression prevented mitochondrial dysfunction and cell death in cultured human proximal tubular cells in a diabetic milieu [182].

INT-767 activates the nuclear hormone receptors, farnesoid X receptor (FXR) and the G protein-coupled receptor TGR5 to confer nephroprotection in mice with streptozotocin–induced T1DM and in T2DM db/db mice (lower proteinuria, podocyte injury, mesangial expansion, and tubulointerstitial fibrosis) through recruitment of multiple pathways, including stimulation of AMPK/SIRT1/PGC-1α [183]. By contrast, the selective FXR agonist obeticholic acid, which is in clinical use for primary biliary cholangitis, did not modulate PGC-1α [183]. Human recombinant Extracellular Superoxide Dismutase (EC-SOD) also activated AMPK/PGC-1α and it reduced albuminuria, mesangial expansion, and interstitial fibrosis in db/db mice [184].

In podocytes from streptozotocin-induced T1DM mice, progranulin maintained mitochondrial homeostasis via SIRT1-PGC-1α-mediated mitochondrial biogenesis and mitophagy [185]. Pyruvate kinase M2 (PKM2) activity was decreased and the PKM2 small-molecule activator TEPP-46 reversed hyperglycemia-induced mitochondrial dysfunction, partially by increasing PGC-1α mRNA in cultured podocytes and in streptozotocin-induced diabetic mice, in whom it reduced albuminuria and diabetic glomerular histological features [186]. In cultured mesangial cells, fenofibrate improved lipotoxicity via activation of AMPK-PGC-1α [187]. Rap1b expression decreased in tubules from human diabetic nephropathy and overexpression of constitutively active Rap1b improved renal tubular mitochondrial dysfunction, oxidative stress, and apoptosis in rats with streptozotocin-induced diabetes, in association with increased C/EBP-β and PGC-1α expression [188].

PGC-1α is functionally regulated by the lncRNA taurine-upregulated gene 1 (Tug1). Podocyte-specific overexpression of Tug1 in T2DM db/db mice rescued PGC-1α expression and that of its transcriptional targets and improved mitochondrial bioenergetics and the biochemical and histological features of diabetic nephropathy. Tug1 interferes with the expression of C/EBP homologous protein (CHOP), an inhibitor of PGC-1α expression and promotes PGC-1α binding to its own promoter [189,190].

**Other forms of CKD.** In kidneys from CKD patients, PGC-1α and PGC-1α-dependent mitochondrial gene expression was downregulated and their expression positively correlated with the glomerular filtration rate and negatively with fibrosis [39]. Fibrosis is a key process in CKD progression, and PGC-1α was downregulated at the mRNA and protein levels in different murine of renal fibrosis, such as Notch transgenic mice and folic acid-induced kidney fibrosis. Indeed, tubule-specific overexpression of PGC-1α reduced fibrosis and restored mitochondrial content in folic acid-induced fibrosis and in Notch transgenic mice [39,121]. In line with in vivo data, TGF-β1 reduced PGC-1α mRNA in Smad3-dependent manner primary human tubular cells [39]. PGC-1α downregulation consistently leads to lipid accumulation and impaired FAO. Indeed, in human and folic acid-induced kidney fibrosis, decreased PGC-1α expression correlated with impaired FAO. FAO inhibition itself has been linked to kidney fibrosis. Thus, pharmacological or TGF-β1-induced inhibition of FAO leads to a profibrotic phenotypic shift characterized by epithelial-mesenchymal transition. This was independent from glucose metabolism, highlighting that FAO itself is a driver of fibrosis [121].

Aldosterone is a strong contributor to podocyte injury and CKD progression, and also induced mitochondrial dysfunction and PGC1a downregulation in podocytes in vivo and in vitro [191]. In fact, both, PGC-1α overexpression or preservation of PGC-1α expression by resveratrol reduced mitochondrial dysfunction and podocyte injury in aldosterone exposed mice [191,192].

## 6. Summary and Future Perspectives

In conclusion, there is increasing evidence for a role of an absolute or relative PGC-1α deficiency on the kidney susceptibility to diverse forms of acute and chronic injury. Indeed, low PGC-1α activity appears to be a common feature of AKI and CKD. The functional impact of PGC-1α has been conclusively demonstrated in preclinical kidney injury as PGC-1α deficiency in either whole kidney or specific kidney cell types, was deleterious while genetic PGC-1α overexpression was generally protective. However, this therapeutic approach is unlikely to reach the clinic. Thus, of even more interest is the characterization of drivers of PGC-1α downregulation that can be targeted therapeutically, including PDE, Notch1, TGF-β1 and TWEAK. Additionally, a host of drugs increases PGC-1α through diverse mechanisms, often through engagement of sirtuins. Advances in the biology and targeting of these pathways are potential avenues to develop PGC-1α activators for clinical use [193,194,195]. Nevertheless, excessive PGC-1α activation may alter mitochondrial homeostasis as observed in podocytes [178], suggesting that it is important to establish the optimal therapeutic window for PGC-1α activation. In addition, PGC-1α over activation could be deleterious in cancer [196,197], thus therapeutic strategies to preserve PGC-1α function should ideally be specific of the tissue/s in which PGC-1α downregulation is pathogenic. 

## Figures and Tables

**Table 1:** Preclinical studies of PGC-1α modulation in AKI.

Model	Treatment	Effect on PGC-1αand Mitochondria	Effect on Kidney Injury		Reference
**Folic acid**	Anti-TWEAK antibodies	↑ PGC-1α ↑ MB		↑ renal function ↓ inflammation	[37]
	Crotonate	↑ PGC-1α		↑ renal function ↓ inflammation	[145]
	Selective cGMP-specific PDE inhibitors	↑ PGC-1α ↑ mitochondrial function ↑ MB		↑ renal function	[34]
	PGC-1α-KO mice	No PGC-1α ↓↓ MB		↓↓ renal function ↑↑ inflammation ↑↑ tubular cell death	[28]
	NMN	↑ Sirt1 ↑ mitochondrial function		↑ renal function	[29]
**Cisplatin**	5-Aminolevulinic acid (even better with Fe)	↑ PGC-1α ↑ mitochondrial function		↑ renal function ↓ tubular cell death	[153]
	PDK inhibitor DCA / PDK4-KO mice	↑ PGC-1α ↑ mitochondrial function ↑ MB		↑ renal function ↓ tubular cell death	[154]
	AICAR / ALCAR	↑ PGC-1α ↑ mitochondrial function ↓mitochondrial fragmentation, ↓ DRP-1		↑ renal function	[155]
**Sepsis**	TLR4-KO / Pharmacologic inhibition of MEK/ERK signaling	↑ PGC-1α ↑ mitochondrial function ↑ MB		↑ renal function	[162]
	STAC (SRT1720)	↑ Sirt1		↑ renal function ↓ inflammation	[167]
	Inducible tubular transgenic mice (iNephPGC1α)	↑ PGC-1α		↑ renal function	[31]
**IRI**	NAM	↑ PGC-1α		↑ renal function ↓ fatty acid accumulation	[31]
	Pharmacologic inhibition of MEK/ERK signaling	↑ PGC-1α ↑ MB		↑ renal function	[174]
	STAC (SRT1720)	↑ PGC-1α ↑ mitochondrial function ↑ MB		↑ renal function ↓ inflammation↓ tubular cell death	[175]
	Caloric restriction	↑ PGC-1α		↑ renal function ↓ inflammation	[176]
	Formoterol	Unchanged PGC-1α ↑ mitochondrial function		↑ renal function	[171]
	PGC-1α-KO mice	Absent PGC-1α		↓↓ renal function ↓↓ mitochondrial function ↑ fatty acid accumulation	[31]
	Inducible tubular transgenic mice (iNephPGC1α)	↑ PGC-1α		↑ renal function ↓ fatty acid accumulation	[31]

Abbreviations: AICAR: 5-aminoimidazole-4-carboxamide-1-β-d-ribofuranoside, ALCAR: antioxidant agent acetyl-l-carnitine, cGMP: cyclic guanosine monophosphate, DCA: dichloroacetate, DRP-1: dynamin-related protein-1, ERK: extracellular signal-regulated kinase, MB: mitochondrial biogenesis, MEK: mitogen-activated protein kinase kinase, NAM: niacinamide, NMN: nicotinamide mononucleotide, PDE: phosphodiesterase, PDK: pyruvate dehydrogenase kinase, Sirt1: sirtuin 1, STAC: sirtuin activating compound, TLR4: toll-like receptor 4, TWEAK: TNF-like weak inducer of apoptosis.
